# Engaging the communities in Wuhan, China during the COVID-19 outbreak

**DOI:** 10.1186/s41256-020-00162-3

**Published:** 2020-07-13

**Authors:** Jie Zhu, Yi Cai

**Affiliations:** 1 Wuhan University School of Law, Wuhan, China; 2 Wuhan University School of Health Sciences, Wuhan, 430072 Hubei China

**Keywords:** Community governance systems, Community engagement, COVID-19, Wuhan

## Abstract

During the early stage of the COVID-19 outbreak in Wuhan, the lockdown of the densely-populated metropolis caused panic and disorderly behavior among its population. Community governance systems (CGSs) were mobilized to lead community engagement to address the challenges and issues brought about by the sudden quarantine measures, still unprecedented in any part of the world during that time. This commentary aims to describe and analyze the roles of the CGSs, its implementation of culturally-tailored strategies and the performance of new functions as called for by the outbreak. We will introduce the community governance structure which has two parallel administrative units of government including the branches of the Communist Party of China (CPC). The pandemic showed that the roles of the CGSs evolved and may continue to be improved in the future. It is important to engage the community and to have community-based approaches in addressing issues brought about by lockdowns. This community experience in Wuhan provides important lessons for the rest of the world.

## Background

Wuhan is a crowded metropolis with a population of 14 million. The city has come to a halt during a metropolitan-wide quarantine on January 23, 2020 due to the spread of COVID-19. Major problems erupted in Wuhan at the early stage of the quarantine. Hospitals were inundated with patients because of the public panic. This resulted in severe shortages of medical supplies placing health care workers at high risk to infection. The epidemic started to overwhelm the health care system. Circulating rumors aggravated the situation which led to trust issues on the government. As problems kept on mounting, Wuhan initiated strict containment strategies at the community level. This meant restricting the movement of the population and strictly confining them within their homes and communities. As a result, normal daily lives of the residents were disrupted including the celebration of the Spring Festival. The quarantine and confinement of the millions of population brought about unique emotional and other demands necessitating public service responses. A community approach was initiated by the government to address the need of the communities by mobilizing the Community Governance Systems (CGSs), a grassroots-level unit of governance instituted across the country. The CGSs was tasked and given the responsibility to meet the various needs of the community, implement containment strategies, and play multiple roles in engaging with the local residents. This commentary aims to describe and analyze the roles played by the CGSs and to show the community dimension of governance during the pandemic in China.

## The community governance system

**Fig. 1:**
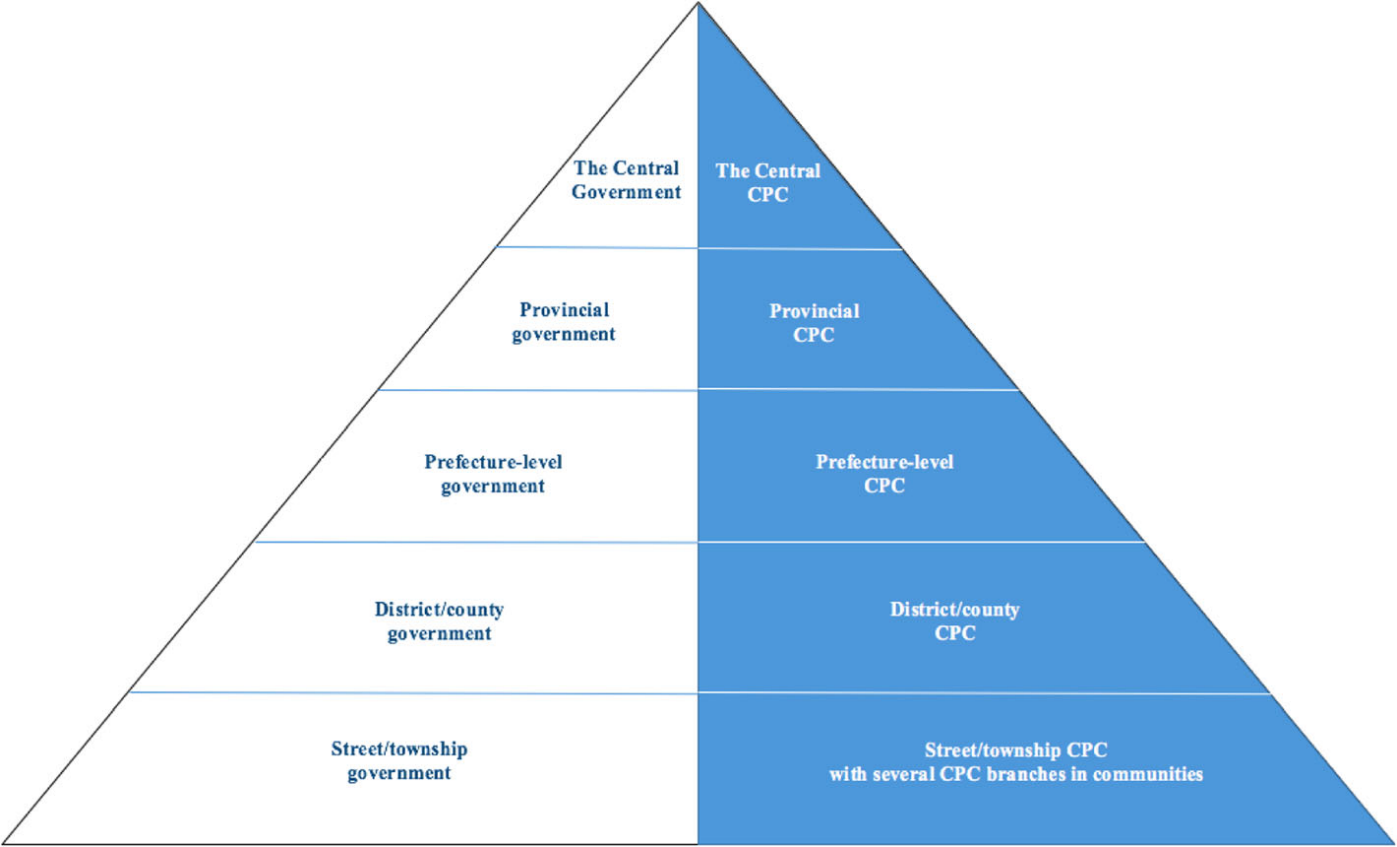
Two parallel administrative units

A community is a small-scale, kinship-, or neighborhood-based unit as compared to a society which pertains to a “large-scale, and competitive market-based unit” [1]. In the context of China, communities are the cells of the Chinese society, usually referred to as “communities” in urban areas and “villages” in rural areas. The Chinese society is administered by a top-down hierarchical governance system, which is composed of five levels of governments, from the central government to the grassroots level of urban street/rural township governments (Fig. 1). The grassroots unit of government plays a role in community governance.

Community governance in China is characterized by the integration of an autonomous governance component juxtaposed with a hierarchical governance component. These components are composed and performed by three types of organizations. The autonomous governance is a function of the homeowner associations. With the development of real estate in China, most urban residents became homeowners. Homeowner associations were then established to manage their own communities. Property management companies are under the management of the homeowner associations. Directors of the homeowner associations are elected by the community residents.

The hierarchical governance is performed by the grassroots or local governments and the branches of the Communist Party of China (CPC). They work hand-in-hand and in parallel with each other. The grassroots government takes charge of community governance to maintain social stability. They are deployed governmental staff who work in the homeowner associations. The Central CPC requires each homeowner association to establish a CPC branch. Members of a CPC branch in the community are residents themselves living in the same community. The CPC branch secretaries are elected by the CPC members within the homeowner associations. The CPC members are deemed to contribute more voluntary work and have to perform their functions and responsibilities to the highest standards as part of the CPC accountability system and code of conduct. On the other hand, there is a lesser demand on the non-CPC members. To illustrate this point, on 16 February, four grassroots government officials who were CPC members were meted disciplinary actions for not being able to meet the standards of work during the containment period [2]. 580,000 CPC members from high-level government offices volunteered for the communities in Wuhan.

## Culturally-tailored CGS community engagement strategies

As a prerequisite for community engagement to the COVID1–9, the World Health Organization (WHO) suggested the use of community influencers. They can be community and religious leaders or health workers [3]. Within the Chinese society, the CPC members are identified to be the most influential and most trusted. During the emergency situation, the CPC members were logically identified as community influencers. They showed effectiveness in mobilizing the people. In addition, CPC members also performed as role models and servant-leaders, which enhanced their ability to influence. They were the first to contribute to the communities which gradually influenced public engagement and in having more volunteers for community services. These volunteers drastically relieved the shortage of human resources needed within the CGSs. Most communities were provided with public services since the CPC have branches and a wide network in most of these areas, thus not missing out on any community. Because the CPC members share The belief of the CPC members on the same guiding principles, which are consistently followed, made the cooperation among them smooth and the functions were performed efficiently and fast.

The CGSs faced challenges in implementing some containment strategies such as staying home, which was difficult to follow during the celebration of the Chinese Lunar New Year when the COVID-19 first broke out. It is customary to celebrate by sharing a meal with family members during the New Year’s Eve, visiting relatives and friends, and hosting parties. Cancelling all festivities, particularly in rural areas, was deemed difficult, if not futile. To address this challenge, the CGSs created many culturally tailored initiatives to improve the understanding of the containment strategies. For example, due to the importance of family culture to Chinese people, the CGSs extended the connotation of “family” from a small-family to a big-family which pertained to the city of Wuhan; even encompassing the forty counties within the area. This made the people easily grasp the concept of solidarity in fighting the battle against the COVID-19. They were observed to be more understanding and cooperative during the latter part of the outbreak.

## Empowering the CGSs with new functions

One of the tasks of the CGSs during the lockdown and the community closure was to establish a referral system for patient triage within their communities. Before patients are transferred to the hospitals, CGSs together with community health workers were tasked to be gate-keepers. They identified and diagnosed suspected patients and transferred confirmed cases to the hospitals. Prior to this, since there was no established protocol for hospital referral, patients would directly go to the hospitals upon experiencing fever [4]. This necessitated the establishment of a referral system and patient triage. The CGSs became responsible for screening febrile patients in the communities and transferring them to quarantine sites for medical observation or sending them to fever clinics for diagnosis [5]. This made the system efficient. On February 12, 2020, for example, over 13,000 cases including clinically diagnosed cases were screened within the communities in 24 h [6].

The CGSs also played a role in health information management by acting as a hub for information transfer. The CGSs collected first-hand health information from the residents and reported it to higher authorities who then collected and summarized all information before being reported back to the public. This contributed to risk communication strategies and in the provision of timely response to public concern.

Ensuring that families remain at home, the CGSs had to assist in providing them services and supporting their various needs. The CGSs organized online grocery shopping, delivered medications to chronic patients, provided transportation to residents who needed medical emergency care, and assisted the police in persuading and enforcing quarantine procedures for non-compliant residents [7].

## Policy implications

Engaging the community and having community-based approaches in addressing COVID-19 have been shown to play a significant role in addressing the issues brought about by the lockdown in the COVID-19 outbreak in Wuhan. Through the community-centered approaches including the mobilization of CGSs, the redefinition of their roles, the use of community influencers, and the employment of culturally tailored strategies, national COVID-19 initiatives became more effective and efficient. This experience in Wuhan may provide lessons for the rest of the world in addressing their local outbreaks.

## Data Availability

Not applicable.

## Abbreviations

COVID-19: Coronavirus disease 2019
CGSs: Community governance systems
CPC: Communist Party of China
